# The time is now: a call for action to translate recent momentum on tackling tropical snakebite into sustained benefit for victims

**DOI:** 10.1093/trstmh/try134

**Published:** 2019-01-21

**Authors:** Robert A Harrison, Nicholas R Casewell, Stuart A Ainsworth, David G Lalloo

**Affiliations:** Liverpool School of Tropical Medicine, Pembroke Place, Liverpool, UK

**Keywords:** Tropical snakebite, World Health Organization, WHO Snakebite Envenoming Working Group

## Abstract

Like the other WHO-listed Neglected Tropical Diseases (NTDs), snakebite primarily affects rural, impoverished tropical communities that lack adequate health resources. The annual 138 000 deaths and 400 000 disabilities suffered by these subsistence farming communities means that snakebite is an additional cause and consequence of tropical poverty. Unlike most of the NTDs, however, snakebite is a medical emergency, and requires rapid treatment in a hospital equipped with effective antivenom, beds and appropriately trained staff. The lack of such facilities in the remote areas most affected by snakebite, and the high treatment costs, explains why most victims, particularly in sub-Saharan Africa, consult traditional healers rather than seek hospital care. Whilst affordable, there is no evidence that traditional treatments are effective. The number of snakebite victims that die, unregistered, in the community is threefold higher than hospital-recorded deaths.

After decades of inertia, WHO benefitted from advocacy interventions and the support of key agencies, including Médecins Sans Frontières, the Wellcome Trust, the Kofi Annan Foundation and the Global Snakebite Initiative, to recently institute transformative actions for reducing the public health burden of tropical snakebite. It is imperative that WHO and the other stakeholders now gain the support and investment of governments, research funders and donor agencies to ensure that this recent momentum for change is translated into sustained benefit to snakebite victims.

## Introduction

Tropical snakebite was a major focus of the 2018 annual meeting of the Royal Society of Tropical Medicine & Hygiene, and the society also launched International Snakebite Awareness Day. These events exemplify the rapid, very recent change in perception of the public health importance of tropical snakebite. Two decades ago, it was society members Theakston and Warrell’s paper entitled ‘Crisis in snake antivenom supply for Africa’^1^ that first reported that tropical snakebite victims are forced to live in a therapeutic vacuum leading to increased rates of mortality and morbidity.

Despite the important message of this and several other academic papers,^2–6^ little changed in the following 15 years to reduce the estimated 138 000 snakebite victims who die each year from respiratory paralysis, hypovolemic shock or uncontrolled bleeding.^6^ To put this into perspective: snakebite globally kills one quarter of the number of people dying from malaria every year; in India, half the number of people dying from HIV are killed by snakebite (46 000 per annum); and more people are killed each month by venomous snakes than the 11 300 people that died during the 2014–2016 West African Ebola crisis. The 400 000 surviving snakebite victims left annually with life-changing physical disabilities^7^ or psychological trauma^8^ add substantially to the disease burden posed by tropical snakebite.

The following sections summarise the circumstances that identify tropical snakebite as a consequence and cause of rural tropical poverty, and why it can be described as perhaps the most marginalised, high-mortality, high morbidity, Neglected Tropical Disease (NTD).

## Snakebite victims typically live in impoverished, remote subsistence farming communities

Poor farmers are dependent upon non-mechanised agricultural techniques that place them at increased risk from snakebite. Peaks in snakebite are often coincident with rain seasons because the fall in temperature and ground-flooding drives venomous snakes into closer contact with farmers just as their agricultural activities increase.^9^ Young adult farmers are therefore the highest risk group for snakebite,^10^ and the loss of their income and food production can drive families into extreme poverty. Inexpensively constructed homes of mud walls and thatched roofs provide little protection against the entry of snakes preying upon rodents attracted to the food, water or chickens within. Inhabitants sleeping on the floor or without bed nets are additionally vulnerable to snakebite at night. Limited electricity supplies also mean that snakes are less visible at dusk/night—a time when they are particularly active. Children are the second highest risk group and are bitten while undertaking chores (e.g. herding) or playing (e.g. catching rats) and other activities less common in more affluent communities. The long-term disability and disfigurement suffered by surviving children frequently results in permanent loss of education and income, and the detrimental effects of stigmatisation. The suspected effects of chronic psychological sequalae upon income and quality of life has yet to be adequately determined.

## Delayed access of victims to effective healthcare

Snakebite is a medical emergency requiring rapid treatment. But the communities at greatest risk are typically located far from effective healthcare and lack access to adequate/reliable ambulatory services. The closest clinic/health centres rarely possess the resources to effectively treat snakebite. Victims therefore often need to travel long, costly distances for several distressing hours before locating a hospital with the requisite clinical capacity. This delay increases the severity of envenoming and likelihood of death. Threefold the number of snakebite deaths recorded from hospital data occur in the community—and these lives lost are therefore not generally recorded.^11^ Cultural circumstances can add to these logistic barriers to accessing effective healthcare. Thus, reports from communities identify that many rural snakebite victims do not seek hospital treatment because the bite is often perceived as a manifestation of witchcraft or deity displeasure,^12^ and that hospital treatment will not reverse these.

## Effective treatment is often unavailable and unaffordable to communities in greatest need, especially in Africa

Antivenom is the drug of choice for treating snakebite and is immunoglobulin purified from the blood of horses/sheep hyperimmunised with snake venom(s). The efficacy of an antivenom is largely restricted to the snake species/genus whose venom(s) were used for immunisation. For example, an antivenom designed to treat cobra envenoming will not be effective against a puff adder envenoming, which limits both the clinical utility and economy-of-scale manufacturing incentives of current antivenoms. The tragedy of sub-Saharan Africa is that the vast majority of available antivenoms (>90% by one estimate) are poorly effective,^13,14^ and some are dangerously ineffective^15,16^ because they are either manufactured with venoms from non-African snakes, or they possess low concentrations of effective immunoglobulins. The Africa-wide distribution success of these inadequate products is primarily because (i) their retail costs (US$30–90/vial^13,14^) are a fraction of that of effective products (US$315/vial for the South Africa vaccine producers SAIMR’s products), and (ii) manufacturers of the most effective African antivenoms (Behringwerke and Sanofi Pasteur) ceased production because of low government demands and commercial inability to out-compete the cheaper but routinely less effective brands.^17^

Effective snakebite treatment typically requires 2–10 vials of antivenom and these costs are very rarely subsidised in Africa. The typical annual income for an African subsistence farmer is US$600–700. Antivenom treatment, if available and effective, is therefore rarely affordable for most African victims. High treatment and transport costs, and poor effectiveness of treatment often leads to a loss of confidence in standard health systems and means that rural snakebite victims often first consult a local, trusted and affordable traditional healer. Unfortunately, while affordable, there is no evidence that traditional treatments are effective.

All of this means that snakebite victims in sub-Saharan Africa have been failed for decades by the absence of adequate responses from governments and international health agencies.

## Raising global awareness and effecting change

It required the intervention of global champions of disadvantaged populations to raise awareness and effect change. This started in 2015 with the widely publicised Médecins Sans Frontières report of the marginalisation of tropical snakebite from the agendas of relevant government and international health agencies.^18^ The Wellcome Trust sponsored a multi-stakeholder ‘Mechanisms to reverse the public health neglect of snakebite victims’ meeting in September 2015 that outlined the challenges and opportunities to address this topic.^19^ In December 2016, Mr and Mrs Kofi Annan convened the important ‘Snakebites in Africa: Challenges and Solutions’ conference that brought together representatives from WHO and from key funding agencies and NGOs along with snakebite clinicians and scientists to identify priority actions.^20^ Following that conference, and with the support of the Kofi Annan Foundation, Médecins Sans Frontières, the Global Snakebite Initiative, Health Action International, and several governments and academics, WHO listed tropical snakebite as a priority NTD in 2017—a decision ratified by the World Health Assembly in April this year.^21^

During the process of this recent transformative change for tropical snakebite, WHO also instituted an antivenom risk-assessment programme designed to identify effective and ineffective brands of antivenoms marketed in Africa. Results of that assessment, including WHO procurement recommendations, are being released in the near future. WHO has also convened a Snakebite Envenoming Working Group (a widely consultative body) of experts to design a strategy to halve the global mortality and morbidity caused by tropical snakebite by 2030. The complexities of this important task and its need for substantive support and investment were made clear by the late Mr Kofi Annan in his ‘Snakebite: The biggest public health crisis you’ve never heard of’ address last June, in which he stated: ‘I strongly believe that snakebite envenoming poses a serious public health challenge. But it is a challenge that can be surmounted. This is a forgotten crisis, and we need to take immediate, robust, and sustained action to confront it. By working together to tackle snakebite, we can save the lives of tens of thousands of our fellow human beings in some of the poorest and most marginalised parts of our world.’^22^

Mr Annan understood the significant barriers faced by tropical snakebite victims, and his global stature and persuasive voice has helped galvanise WHO engagement with key health decision-makers in governmental, international and philanthropic donor agencies. There is an urgent need to utilise this new momentum to garner substantive global investment in a number of actions at every stage in the snakebite victims’ route to recovery:

- Delivery of community education campaigns to reduce the incidence of snakebite and to promote the accessing of hospital care.
- Investment in capacity strengthening of local ambulance and health infrastructures so that victims have rapid access to health facilities equipped with effective antivenoms, and investment in training of clinical staff.
- Establishment of regional antivenom-efficacy testing centres to ensure that inappropriate antivenoms are excluded from distribution.
- Urgent investment in clinical trials of existing and new antivenoms, particularly in Africa.
- Engagement of governments and other stakeholders with manufacturers and distributors of effective antivenoms to ensure a substantial increase in production and a regular supply of their outputs at affordable prices.
- Establishment of in-country surveys to accurately identify where snakebite incidence, mortality and morbidity is greatest, and thereby improve antivenom delivery to priority areas.
- Scientific support for the development of new products and diagnostic tools that are affordable, effective against all regional snakes and safe enough to be used in the community.

These actions will require support from many different individuals and agencies. However, global and sustained change requires a coordinated response, and WHO with its many supporters is perhaps the only agency with the requisite reach and influence. Donor agencies need to provide WHO with the investment it needs to implement its strategic plan. Effective delivery of this combination of outputs will substantially and sustainably reduce tropical snakebite as a global health concern and demonstrate that the health needs of the world’s snakebite victims are no longer neglected.
